# Effect of Polydopamine-Coated Strontium-Doped Hydroxyapatite Nanowires on Bone Marrow Mesenchymal Stem Cells and Umbilical Vein Endothelial Cells

**DOI:** 10.3390/polym17081039

**Published:** 2025-04-11

**Authors:** Hanjing Li, Yucheng Liu, Longhai Peng, Chunyuan Du, Kui Zhou

**Affiliations:** 1 School of Advanced Manufacturing, Nanchang University, Nanchang 330031, China; 2 Key Laboratory of Immune Microenvironment and Disease (Ministry of Education), Tianjin Key Laboratory of Cellular Homeostasis and Disease, Department of Physiology and Pathophysiology, School of Basic Medical Sciences, Tianjin Medical University, Tianjin 300070, China; 3 Nanchang Municipal Key Laboratory of 3D Bioprinting Technology and Equipment, Nanchang University, Nanchang 330031, China

**Keywords:** hydroxyapatite nanowires, polydopamine, strontium, cell spheroid

## Abstract

Hydroxyapatite nanowires (HAW) can effectively improve the bone repair ability in bone engineered tissue. However, due to their single function, the application of HAWs in biological tissue engineering materials is limited. In this study, strontium-doped hydroxyapatite nanowires (SrHAW) were synthesized by a hydrothermal method and coated with polydopamine (PDA) to improve the function of HAWs. The material structure, biocompatibility evaluation, and differentiation capability testing of PDA-coated strontium-doped hydroxyapatite (SrHAW@PDA) nanowires were conducted. Then, the nanowires were co-cultured with rat bone marrow mesenchymal stem cells (BMSCs) and rat umbilical vein endothelial cells (UVECs) to prepare cell spheroids. Compared with the undoped and uncoated HAW, the SrHAW@PDA nanowires enhanced the cell activity and their angiogenesis and osteogenesis abilities. In addition, their performance in the three-dimensional spheroid also played a positive role in the cells in the spheroid. Due to the presence of PDA, the adhesion between the cells in the three-dimensional spheroid and the nanowires were enhanced. In summary, these results show that SrHAW@PDA has the potential to be used as an alternative material to regulate cell biological activity in three-dimensional cell spheroids.

## 1. Introduction

In recent years, much research has focused on using the power of functional biomaterials to improve tissue regeneration and repair [1,2]. To successfully carry out bone tissue regeneration, appropriate materials and parameters need to be selected to simulate the complex bone microenvironment. Hydroxyapatite (HAp), as the fundamental component of bone mineral, exhibits excellent biocompatibility and a chemical composition like that of natural bone. In these applications, hydroxyapatite exhibits diverse structures, such as spherical, rod-shaped, and nanowire forms [3,4,5]. For example, HAp is mineralized and combined with stem cells to design a complex aggregate osteochondrogenic microenvironment [4]. Hydroxyapatite nanowires (HAWs) are loaded with iron ions and coated with PDA to promote nerve repair [3]. Nanomaterials regulate cell fate by modulating the mechanical microenvironment, controlling the release of growth factors, and providing a specific cell–material interface [6,7]. Due to the influence of size, structure, and the cell–material interface, nanostructures can be used to stimulate proteins on the cell membrane surface and release inorganic ions to regulate cell fate [8]. Our previous work has confirmed that, as a building block for engineered bone tissue, HAW within three-dimensional spheroids release ions that promote osteoblast-related cells, demonstrating accelerated bone repair capabilities. This primarily depends on the combination of nanowires with cell spheroids and the release of Ca^2+^ ions within them [9]. Uniformly mixed small-sized nanomaterials effectively transmit osteogenic signals to cells in three-dimensional spherical structures, and the results show an enhanced osteogenic differentiation ability. However, there are still limitations in maintaining cell viability within aggregates and stimulating vascularization during bone regeneration.

The element doping of bionanomaterials can modulate the crystal structure and physicochemical properties of the materials [10,11,12], and can play a guiding role in determining cell fate in tissue engineering. Many studies have shown that the addition of bioactive chemical elements, such as silicon, copper, strontium, etc., can promote osteogenesis and angiogenesis [13,14]. Various materials incorporating Sr, including bioceramic scaffolds [15], bone cement [16], and bioactive glass [17], have been developed and applied in bone tissue engineering. It has been reported that the degradation products of strontium-doped calcium polyphosphate enhance the adhesion and angiogenic behavior of endothelial cells (ECs) [18]. Strontium ranelate (SrR), whose main component is strontium ions, has been clinically proven to effectively promote bone reconstruction and vascular formation, and is effective against postmenopausal osteoporosis in women [19]. It has recently been found that strontium ions may influence angiogenesis [20]. Cells cultured in the extract medium containing strontium ions can enhance the expression of angiogenesis-related genes [21]. Therefore, it can be hypothesized that incorporating Sr into hydroxyapatite nanowires may combine osteogenic and angiogenic properties to achieve the goal of vascularized bone regeneration.

Surface coating technology can provide special sites for cells and proteins, thus promoting cell biological properties [22]. The surface morphology mainly affects the tension of the cytoskeleton and then changes the shape of the cell, which determines the cell’s fate [22]. Chemical signals can provide cell recognition sites, such as Arg-Gly-Asp [23], to enhance cell adhesion or connect biological factors to guide cell differentiation [24,25]. Mussel-inspired PDA can be spontaneously attached to any complex surface with a high binding strength [26,27]; the relatively rough surface morphology of the carrier contributes to a better interaction between cells and materials [28]. The surface coated with PDA provides a suitable breeding ground for most mammalian cell lines to adhere and spread [29]. Wang et al. [30] confirmed that the PDA layer can promote the adhesion and proliferation of chondrocytes on the surfaces of poly(ε-caprolactone) (PCL), poly (L-lactic acid) (PLLA), and polyurethane. Catechol of PDA can attract and bind bioactive components and metal ions to induce hydroxyapatite deposition [31]. Most importantly, a PDA coating can stimulate osteogenic differentiation. Angiogenesis is closely related to bone regeneration and plays an indispensable role in regulating hard tissue regeneration and balance in vivo [32].

During the process of bone formation, the recruitment of biological factors and cells is crucial, and angiogenesis is one of the prerequisites for new bone formation and remodeling in bone repair [33]. The use of PDA can attract and bind biological factors and metal ions, induce HAP deposition, and enhance cell survival. Additionally, Sr ions can promote the angiogenic capacity of cells. The purpose of this study was to investigate the ability of SrHAW@PDA to maintain cell activity, osteogenesis, and angiogenesis. Considering the requirement for excellent biocompatibility, we selected hydroxyapatite [Ca_10_(PO_4_)_6_(OH)_2_(HA)] as the carrier, incorporated strontium ions, and coated them with PDA to prepare strontium-doped hydroxyapatite coated with PDA (SrHAW@PDA). BMSCs and UVECs were used as three-dimensional cell models to evaluate the effects of strontium doping and PDA coating on the interaction between BMSCs and UVECs.

## 2. Materials and Methods

### 2.1. Fabrication of SrHAW@PDA

**Figure 1:**
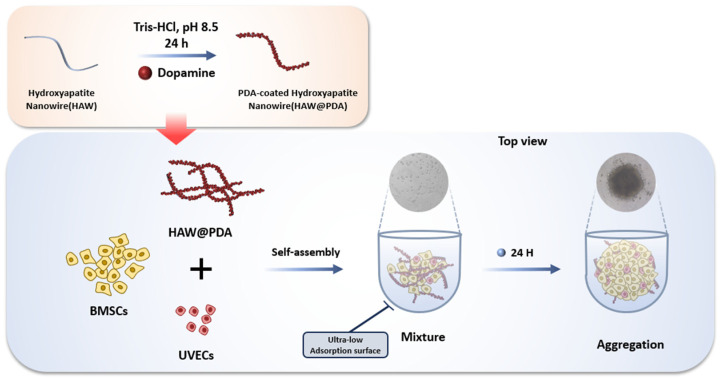
Schematic diagram of material synthesis and cell culture.

In this experiment, HAW were prepared by a hydrothermal method using a calcium oleate precursor solvent, as previously reported [34]. For the synthesis of HAW containing 4% of the element Sr, strontium chloride hexahydrate (SrCl_2_·6H_2_O) was added in the process instead of CaCl_2_ to synthesize 4SrHAW with the molar ratio of Sr/(Ca+Sr) at 4%. For SrHAW@PDA, 20 mg SrHAW was added to Tris-HCl buffer solution (10 mM, pH = 8.5) and stirred for 10 min. Then, dopamine (20 mg, Sigma-Aldrich) was added to the solution and the mixture was stirred in the open air for 24 h. SrHAW@PDA was collected after centrifugation with 6000 rpm for 5 min. HAW, SrHAW, and SrHAW@PDA were washed with DI water for future cell culture (Figure 1).

### 2.2. Material Characterization of Nanowires

X-ray powder diffraction patterns were recorded using an X-ray diffractometer (XRD; Bruker, Billerica, MA, USA). Fourier transform infrared spectra were obtained on an FTIR spectrometer (FTIR; Thermo Scientific, Waltham, MA, USA). Scanning electron microscopy images were obtained using scanning electron microscopes (SEM; FEI NanoPorts, Hillsboro, OR, USA). Transmission electron microscopy images were obtained using a transmission electron microscope (TEM; FEI NanoPorts, Hillsboro, OR, USA).

### 2.3. Cell Culture

BMSCs were obtained from Wuhan Procell Life Technology (Wuhan, China) and cultivated in Dulbecco’s modified Eagle’s medium (DMEM/F12, MULTICELL, Dallas, TX, USA) with 1% P.S. and 10% fetal bovine serum. UVECs were obtained from Shanghai Fuheng Biotechnology (Shanghai, China) and cultured in ECM medium (ScienCell, 1001, Carlsbad, CA, USA) containing 1% P.S. and 10% FPS. The medium was maintained in an incubator at 37 °C with a 5% CO_2_ atmosphere. The 3–4 passages of BMSCs and 3–7 passages of UVECs were used in subsequent experiments.

### 2.4. Cell Proliferation Assay and Alkaline Phosphatase (ALP) Activity Assay

To qualitatively and quantitatively evaluate the biological activity of nanowires, Live/Dead staining and a CCK-8 assay were carried out, respectively. A total of 4000 cells (BMSCs: UVECs = 92:8) were co-cultured with 1 μg HAW, 4SrHAW, and 4SrHAW@PDA in the well plate, while 4000 cells without materials were cultured in the Control group. For Live/Dead staining, 2D co-culture samples were cultured for 7 days. The medium was replaced by 100 μL PBS containing 1 μL Calcein AM and 1 μL propidium iodide and was incubated at 37 °C for 30 min. The sample was visualized by a fluorescence inversion microscope (Nikon, Tokyo, Japan). For the CCK-8 assay, 2D co-culture samples were cultured in 96-well plates, and the medium was replaced with 100 μL growth medium and 10 μL CCK-8 solution. After incubation at 37 °C for 2 h, the absorbance of each sample was measured at 450 nm using an enzyme labeling instrument (Thermo, Multiscan MK3, Waltham, MA, USA). The OD values were obtained and statistically analyzed. For ALP staining, the cell suspensions of BMSCs and BMSCs + UVECs co-cultured with HAW, 4SrHAW, and 4SrHAW@PDA for 24 h were used. The cells were then cultured under osteogenic conditions for 7 days. According to the manufacturer’s instructions, an ALP staining kit (Beyotime Biotechnology, C3206, Shanghai, China) was used to evaluate the ability of HAW to induce osteogenic differentiation.

### 2.5. Tube Formation Assay

The tube formation experiment was performed to evaluate the angiogenic ability of nanowires on UVECs in vitro. According to the manufacturer’s instructions, the 96-well plate was coated with 50 μL Matrigel (Beyotime Biotechnology, C0372, Shanghai, China). A total of 2 × 10^4^ UVECs were resuscitated in the cell medium containing 5 μg HAW, 4SrHAW, and 4SrHAW@PDA, respectively, then all experimental groups were inoculated on Matrigel for 0–4 h. At each time point, the cells cultured on Matrigel were observed and photographed with an inverted optical microscope (Nikon, Tokyo, Japan).

### 2.6. Formation and Proliferation of 3D Cell Spheroids with BMSCs, UVECs, and SrHAW@PDA Nanowires

BMSCs and UVECs were harvested with trypsin and collected by 1600 rpm centrifugation for 5 min to prepare spheroids. The mixture ratio of UVECs and BMSCs was 92:8 [35]; on this basis, 1 μg HAW, 4SrHAW, and 4SrHAW@PDA were added to the cell suspension, and each spheroid contained a total of 4000 cells. A 200 μL cell suspension containing nanowires was inoculated into each well of a 96-well plate (Corning, New York, NY, USA) to reach about 4000 cells/spheroid. The cells were maintained in a mixed growth medium (growth medium of 75% BMSCs and 25% UVECs) [36]. After inoculation, the cells were incubated overnight at 37 °C to form spheroids.

The harvested spheroids were observed and imaged under an inverted microscope (Nikon ECLIPSE Ts 2, Tokyo, Japan) in the bright field of vision. Then, the ImageJ software 1.53 (NIH Freeware) was used to quantify the diameter distribution. To qualitatively and quantitatively evaluate the activity of composite spheroids, EdU staining was conducted according to the instructions of the BeyoClick EdU Cell Proliferation Kit (Beyotime Biotechnology, C0071S, Shanghai, China). Samples were observed using laser scanning confocal fluorescence microscopy (LSCM, Nikon, Tokyo, Japan) with Alexa Fluor 488 (EdU) and 405 (DAPI). After composite spheroids were cultured for 7 days, the Cell Counting Kit-3D assay (CCK-3D) was used to treat the samples. The spheroid samples were cultured in 96-well plates, and the medium was replaced with 100 μL of growth medium and 10 μL of CCK-3D solution. After incubating for 2 h at 37 °C, the absorbance of each sample was measured at a wavelength of 450 nm using a microplate reader (Thermo, Waltham, MA, USA). Three parallel samples were analyzed, and an average value was obtained.

### 2.7. Statistical Analysis

Statistical analysis was performed using Origin, and the values are presented as mean ± standard deviation. The sample size (n) for the statistical details of the different situations can be found in the relevant captions. Statistical analyses of protein expression and fluorescence intensity were performed using ImageJ software. The two groups were compared using Student’s *t*-test and one-way ANOVA (one independent variable). Differences were defined as * *p* ≤ 0.05, ** *p* ≤ 0.01, *** *p* ≤ 0.001, and **** *p* ≤ 0.0001, and considered significant at * *p* ≤ 0.05.

## 3. Results and Discussion

### 3.1. Characterization of SrHAW@PDA Nanowires

**Figure 2:**
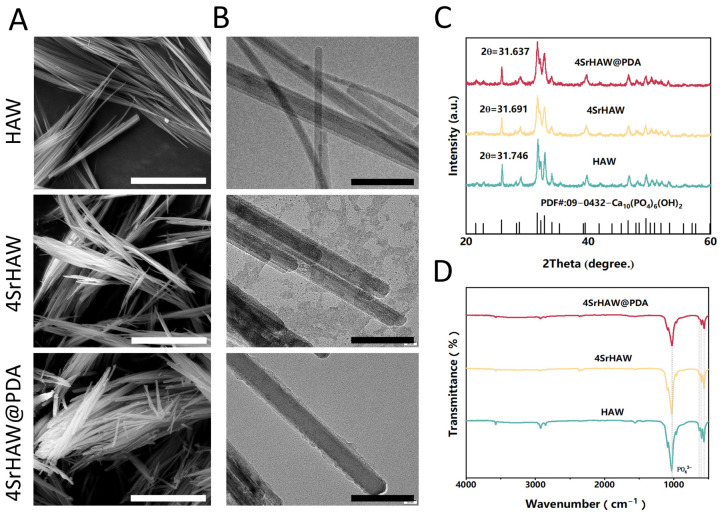
Material characterization of HAW, 4SrHAW, and 4SrHAW@PDA nanowires. (**A**) SEM images of HAW, 4SrHAW, and 4SrHAW@PDA (scale bar = 5 μm). (**B**) TEM images of HAW, 4SrHAW, and 4SrHAW@PDA (scale bar = 100 nm). (**C**) XRD analysis of HAW, 4SrHAW, and 4SrHAW@PDA. (**D**) FTIR analysis of HAW, 4SrHAW, and 4SrHAW@PDA.

It is observed by SEM that HAW, 4SrHAW, and 4SrHAW@PDA nanowires show clusters of nanowires (Figure 2A). Among them, the adhesion of nanowires appears after 4SrHAW is coated with PDA, which may be caused by the stickiness of PDA. Nanowires with a diameter between 1~2 μm are shown in TEM. A layer of 10 nm PDA is attached to the surface of 4SrHAW@PDA nanowires, and the thickness is uniform. The nanowires with a high aspect ratio give HAW the property of easy aggregation (Figure 2B). Fourier transform infrared spectroscopy (FTIR) shows that HAW is representative of the vibrational spectrum of hydroxyapatite, including the peaks around 578 cm^−1^ and 1028 cm^−1^ (representing the characteristic peak of PO_4_^3−^), in which the absorption peak at about 3570 cm^−1^ comes from the water adsorbed in the sample (Figure 2C). X-ray diffraction (XRD) patterns confirm that the prepared HAW, 4SrHAW, and SrHAW@PDA still show a specific crystal phase of HA [Ca_10_(PO_4_)_6_(OH)_2_] [37]. The incorporation of strontium corresponds to the decrease in 2θ values, in which the 2θ values of HAW, 4SrHAW, and 4SrHAW@PDA are 31.746, 31.691 and 31.637, respectively. The results of XRD show that the doping of the strontium element does not affect the phase (Figure 2D).

### 3.2. SrHAW@PDA Improved the Biological Performance of BMSCs and UVECs

**Figure 3:**
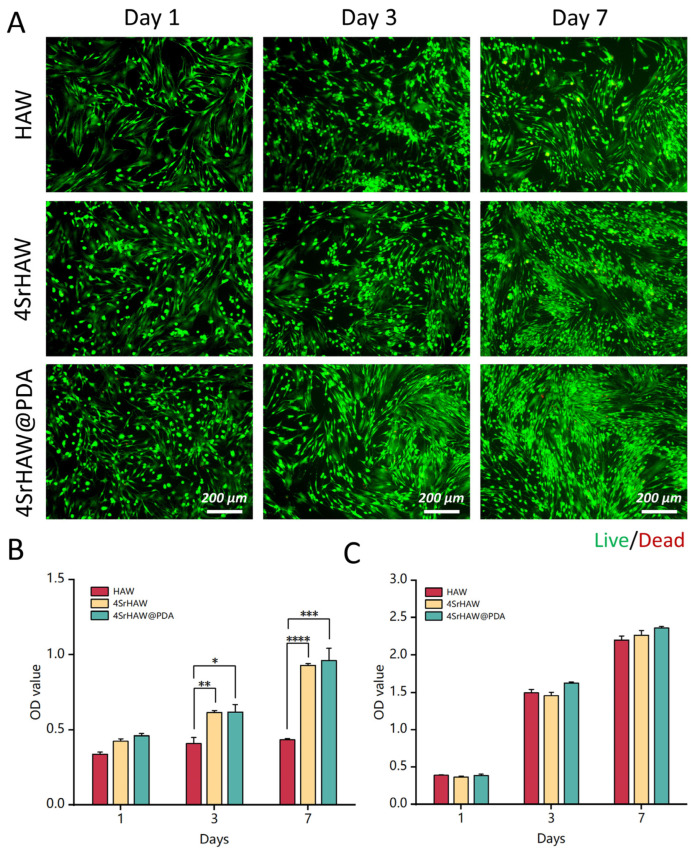
Biocompatibility of HAW, 4SrHAW, 4SrHAW@PDA nanowires. (**A**) Live/Dead staining of BMSCs co-cultured with HAW, 4SrHAW, and 4SrHAW@PDA on days 1, 3, and 7 (scale bar = 200 μm). (**B**) The CCK-8 of BMSCs co-cultured with HAW, 4SrHAW, and 4SrHAW@PDA was measured on days 1, 3, and 7 (one-way ANOVA; * *p* ≤ 0.05,** *p* ≤ 0.01, *** *p* ≤ 0.001, **** *p* ≤ 0.0001). Data are presented as mean ± SD (n = 5). (**C**) The CCK-8 of UVECs co-cultured with HAW, 4SrHAW, and 4SrHAW@PDA was measured on days 1, 3, and 7. Data are presented as mean ± SD (n = 5).

To evaluate the biotoxicity of nanowires to BMSCs and UVECs, BMSCs and UVECs with a certain proportion of HAW, 4SrHAW, and 4SrHAW@PDA were co-cultured. After 7 days of co-culture, the samples were subjected to Live/Dead staining and photographed. In the Live/Dead staining pictures, it can be observed that BMSCs in the HAW and 4SrHAW experimental groups exhibited partial cell aggregation on the third and seventh days of co-culture, displaying clusters of green fluorescence. This phenomenon was particularly pronounced in the HAW group without the incorporation of Sr elements, whereas the cells in the 4SrHAW@PDA group displayed a normal, healthy growth pattern characterized by a whirlpool-like distribution (Figure 3A). This difference may have been related to the structure and surface functional groups of HAW, which was similar to a previous study [38].

In the CCK-8 assay, the growth rate of cells in the HAW group increased slowly, while the growth rate of the other two experimental groups was similar (Figure 3B), which was consistent with the results of the Live/Dead staining experiment (Figure 3A) when co-cultured with BMSCs and with each group of materials. After 3 and 7 days of culture, the cell viability of the 4SrHAW group and 4SrHAW@PDA group was significantly higher than that of the HAW group. In addition, the cell viability of the 4SrHAW@PDA group was higher than that of the two experimental groups without PDA, but there was no significant difference between 4SrHAW@PDA and 4SrHAW. The toxic effect of the material on UVECs was studied, and the activity of UVECs was investigated by CCK8 assay (Figure 3C). During the one week of co-culture, the number of surviving cells in the three experimental groups was similar, but the viability of UVECs in the 4SrHAW@PDA group was higher than that in the HAW group and 4SrHAW group without PDA, but there was no significant difference. The above results show that the biocompatibility of 4SrHAW was improved after PDA treatment. In the CCK-8 assay with UVECs co-cultured with each group of materials, the lack of significant differences in cell viability between the PDA-coated 4SrHAW@PDA experimental group and the two non-PDA-coated experimental groups may have been related to the high proliferation of ECs.

**Figure 4:**
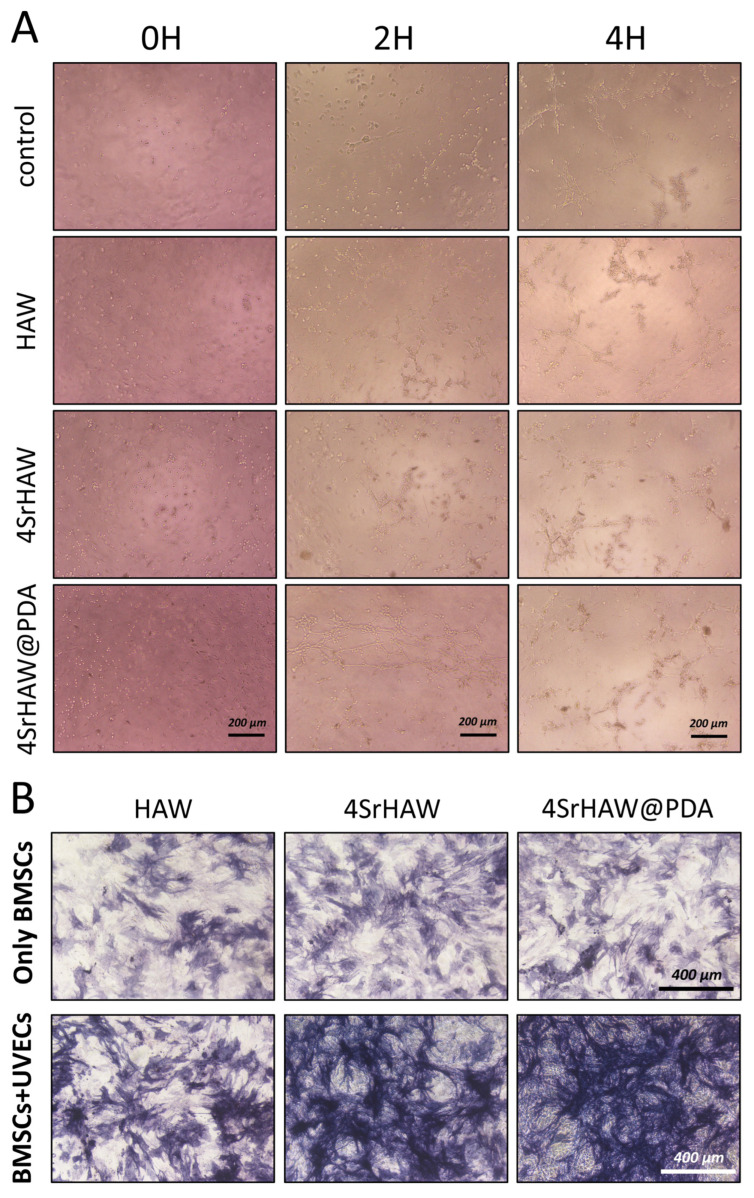
(**A**) Tube formation assay of UVECs co-cultured with HAW, 4SrHAW, and 4SrHAW@PDA for 0, 2, and 4 h (scale bar = 200 μm). (**B**) ALP staining of only BMSCs and BMSCs + UVECs co-cultured with HAW, 4SrHAW, and 4SrHAW@PDA at day 7 (scale bar = 400 μm).

It has been reported that the angiogenic ability of biomaterials can be detected by the ability to promote tubule formation when co-cultured with endothelial cells or other cells [5]. In this study, the tube formation assay was employed to evaluate the tube-forming ability of UVECs when co-cultured with three types of biomaterials: HAW, 4SrHAW, and 4SrHAW@PDA. This capability serves as a crucial indicator for assessing the angiogenic potential of ECs [39,40]. During the co-culture period of 2–4 h, the Control group containing only UVECs exhibited significantly fewer tubes and nodes compared to the three experimental groups. The HAW and 4SrHAW groups formed fewer meshes and tubes than the 4SrHAW@PDA group (Figure 4A). Considering the ability of PDA to absorb macromolecules [41,42], it is possible that PDA absorbed macromolecules from the culture medium that promoted the angiogenesis of UVECs, thus enhancing the angiogenic ability of PDA. However, the specific mechanisms underlying this phenomenon remain unclear. During 2–4 h, the number of nodes and tubules formed in the 4SrHAW group and 4SrHAW@PDA group, which incorporated Sr, was greater than that in the HAW group, with the 4SrHAW@PDA group showing more pronounced results. This outcome was consistent with the findings from the UVEC proliferation assay.

ALP is an important indicator of the osteoblast differentiation of BMSCs. In the early stage of osteogenic differentiation, the protein expression of ALP will continue to increase within 5–14 days [5]. To explore the effects of strontium incorporation, PDA surface coating, and the involvement of UVECs on the osteogenic differentiation of BMSCs, ALP staining was conducted on biological samples after 7 days of co-culture, specifically, on the cell suspension of BMSCs and BMSCs + UVECs co-cultured with HAW, 4SrHAW, and 4SrHAW@PDA (Figure 4B). In the various BMSC experimental groups without the addition of UVECs, the depth of ALP staining was relatively similar. However, the HAW experimental group exhibited slightly lighter staining compared to the other groups. The ALP staining intensity in the three experimental groups with added UVECs was higher than that in the groups containing only BMSCs. In the co-culture of BMSCs and UVECs, both cells showed enhanced osteogenic and angiogenic potential, such as the up-regulated gene and protein expression of ALP, BMP-2, VEGF, and platelet endothelial cell adhesion molecules [43]. The paracrine signal transduction of ECs may contribute to the evaluation of the observed phenomenon by secreting the biological factors of MSC recruitment, proliferation, and osteogenesis [44]. Furthermore, on the seventh day of co-culture of BMSCs and UVECs, the ALP staining intensity in the two experimental groups incorporating strontium was higher than that in the HAW group. This phenomenon was not observed in the three experimental groups without UVECs, indicating that the Sr incorporation and PDA coating promoted the osteogenic differentiation of BMSCs with the presence of UVECs. Many studies have shown that ECs can regulate the differentiation of MSCs [45], including the enhancement of the osteogenic phenotype [46]. At the same time, the nano-morphology matrix with PDA coating can recruit biological macromolecules from the culture medium and connect more integrins to effectively improve osteogenic differentiation [41,42]. In addition, strontium can promote angiogenesis, up-regulate the expression of hypoxia-inducible factor-1 α (HIF-1 α), and enhance the expression of angiogenesis-related genes [47]. Strontium ions can also improve the ability of bone formation and VEGF expression in vitro and in vivo [48,49]. Yu Zhuang et al. demonstrated that cultured cells in the medium containing strontium ions can enhance the expression of angiogenesis-related genes [5].

### 3.3. Effect of SrHAW@PDA Nanowires on Cell Spheroid Formation

**Figure 5:**
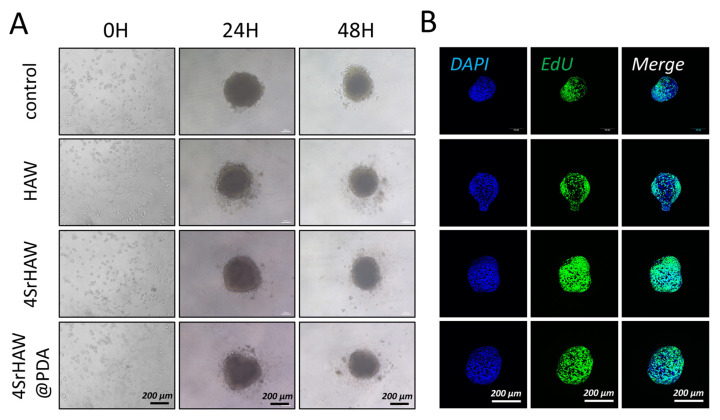
(**A**) Bright field images of cell spheroid assembly process of BMSCs and UVECs with HAW, 4SrHAW, and 4SrHAW@PDA at 0, 24, and 48 h (scale bar = 200 μm). (**B**) EdU staining of cell spheroids of different compositions after 5 days of assembly (scale bar = 200 μm).

We utilized a commercially available U-shaped-bottom 96-well plate coated with a layer of ultra-low-adhesion hydrogel. BMSCs and UVECs were mixed with HAW, 4SrHAW, and 4SrHAW@PDA in the well plate, respectively. Within 24 h, uniform-size and regularly shaped composite spheroids composed of different components were successfully harvested (Figure 5A).

**Figure 6:**
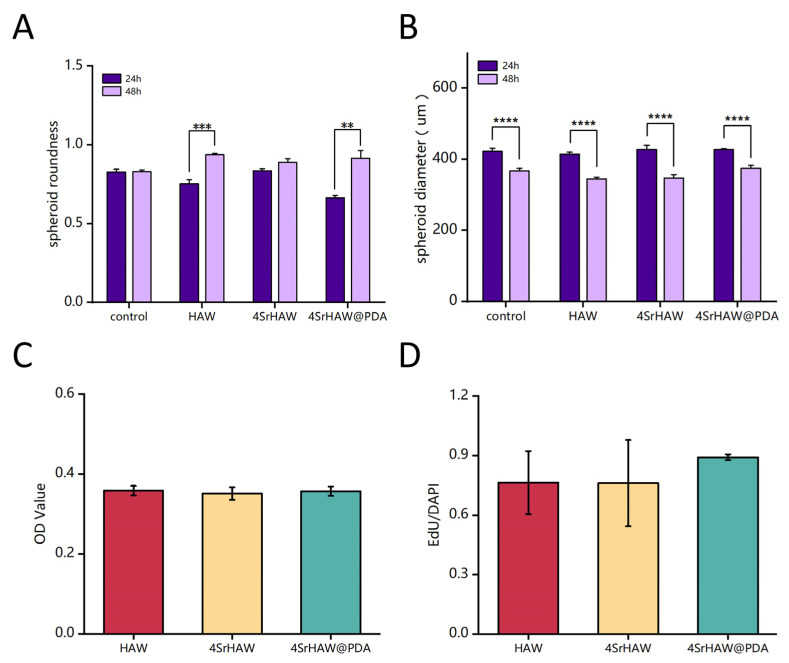
(**A**) Analysis of spheroid roundness after 24 and 48 h of preparation of cell spheroids with HAW, 4SrHAW, and 4SrHAW@PDA (one-way ANOVA; ** *p* ≤ 0.01, *** *p* ≤ 0.001). Data are presented as mean ± SD (n = 10). (**B**) Analysis of spheroid diameter at 24 and 48 h of preparation of cell spheroids with HAW, 4SrHAW, and 4SrHAW@PDA (one-way ANOVA; **** *p* ≤ 0.0001). Data are presented as mean ± SD (n = 12). (**C**) CCK-3D analysis of cell spheroids with HAW, 4SrHAW, and 4SrHAW@PDA on day 7 after preparation. Data are presented as mean ± SD (n = 5). (**D**) Quantitative analysis of EdU-positive cells (one-way ANOVA). Data are presented as mean ± SD (n = 3).

The most direct way to evaluate the effect of nanomaterials on the interior of cell spheroids is to observe their morphological changes [50]. After mixing for 24 h, the cells and materials formed a regular spheroid and the size of the composite spheroid changed with the addition of different components of nanowires (Figure 6A), from ≈422.21 ± 8.46 μm (Control) to ≈427.16 ± 2.97 μm (4SrHAW@PDA). This result should have been related to the scaffold effect produced by nanowires. Different components of nanowires were doped in cell spheroids to avoid too-close bonding between cells. During the 48 h culture time, due to actin stress rearrangement, the spheroid size of the 4SrHAW group changed the most, being reduced by 18.7%. The HAW group experienced a 16% reduction, while the Control group and the 4SrHAW@PDA group showed a smaller decrease of only 12% to 13%. All groups showed significant diameter differences between 24 and 48 h. The incorporation of nanowires with different components led to varying degrees of size changes in the composite spheroid, indicating that the inclusion of nanowires affected the intercellular interactions and cellular reorganization within the cell spheroid.

The aggregates formed by cell self-assembly produced contractile force through the repeat of cellular actin stress fibers and induced spheroid compaction and maturation [51]. Unlike the spheroids formed by single cells in previous studies [38], the roundness of the spheroids formed by the two kinds of cells was not very high. During the 24–48 h after the formation of spheroids, the roundness of the spheroids formed solely by BMSCs and UVECs was even lower than that of some experimental groups with added nanowire materials (Figure 6B). During the 24 h, the morphology of the composite spheroid in the three experimental groups was unstable during the self-assembly process after the material addition, resulting in irregular roundness across the groups. The roundness of the Control group and the three experimental groups was 0.83 ± 0.02, 0.75 ± 0.03, 0.864 ± 0.01, and 0.66 ± 0.01, respectively. With the increase in time, the secretion of proteins such as extracellular matrix (ECM) would make the shape of the spheroid stable. After 48 h of spheroid formation, the roundness of the simple cell spheroid group (Control) was lower than that of the three experimental groups. The roundness of the Control and the three experimental groups were 0.83 ± 0.01, 0.94 ± 0.01, 0.89 ± 0.02, and 0.92 ± 0.02, respectively. The roundness of the Control group increased by only 3.56%, while the 4SrHAW group increased by 7%, the HAW group increased by 24.43%, and the 4SrHAW@PDA group showed a significant increase of 38.00%. Cells possess the ability to reorganize and separate through a process called “cell sorting”, enabling them to form structures that closely resemble the characteristics of tissues in vivo [52]. Compared with the spheroid containing only MSCs, the spheroid composed of MSCs and UVECs showed a denser and more condensed structure with a uniform cell distribution. This phenomenon was primarily observed in the center of the spheroid, with a surrounding ring-like structure at the periphery, which was due to the presence of ECs in the cell spheroid [53]. This showed that the two kinds of cells in the spheroid would have a stronger cell rearrangement, resulting in a greater change in the shape of the spheroid.

It is worth mentioning that the roundness of the spheroids formed by the combination of PDA-coated nanowires and two types of cells was higher than that of the experimental group without PDA-coated nanowires, and the difference was significant. The interaction between cells and materials began with cell attachment, which was essential for cell proliferation and differentiation [54]. The PDA coating could transform the hydrophobic surface into a hydrophilic surface, facilitating protein adhesion and enhancing cell adhesion [55]. In addition, the rich functional groups contained in PDA-coating could effectively absorb proteins and peptides in the culture medium and promote the biological properties of cells [56]. Therefore, this outcome benefited from the PDA-enhanced adhesion between cells and nanowires in three-dimensional cell spheroids, allowing for more thorough reorganization between the two kinds of cells and nanowires. As a result, the composite spheroid of the 4SrHAW@PDA group exhibited higher roundness. Additionally, the compaction of the cell spheroid leading to a reduction in size was a direct factor influencing the surface tension of the cell spheroid. This meant that after the same duration of culture, smaller cell spheroids exhibited a greater ratio of elasticity (solid) to viscosity (liquid), resulting in correspondingly enhanced mechanical properties [57]. Based on maintaining the same cell number and types, the recombination of cells with different components of nanowires in the spheroid led to an increase in roundness, which may have enhanced the mechanical properties of the composite spheroid.

Thymidine analog 5-ethynyl-2′- deoxyuridine (EdU) was used to analyze the cell proliferation in the composite spheroids of the HAW group, 4SrHAW group, and 4SrHAW@PDA group after 7 days of assembly (Figure 5B). EdU was incorporated during DNA synthesis and then marked with Alexa Fluor 488 (green) [57] by click reaction. After seven days of assembly, the BMSCs and UVECs in the composite spheroid proliferated evenly, and the proportion of EdU cells was more than 75% in each group. This may have been related to the robust proliferation viability of both cell types in the spheroid structure. The proportion of EdU-positive cells in the composite spheroid of the HAW group, 4SrHAW group, and 4SrHAW@PDA group was 0.76 ± 0.16, 0.76 ± 0.22, and 0.89 ± 0.01, respectively. The proportion of EdU-positive cells in the two experimental groups (Figure 6D) without PDA coating was higher than that in the 4SrHAW@PDA group. The WST series chromogenic substrates in the Cell Counting Kit-3D kit (CCK-3D) were utilized, with the assistance of an electron coupling reagent, to measure the concentration of dehydrogenase within the mitochondria of cells, thereby assessing the cell viability (Figure 6C) in the composite spheroids of each group on the seventh day. The OD values of the HAW group, 4SrHAW group, and 4SrHAW@PDA group were 0.358 ± 0.012, 0.351 ± 0.016, and 0.357 ± 0.012, respectively. There was no significant difference between the groups. This finding is consistent with the EdU staining results. The cell viability of the experimental group coated with PDA was stronger than that of the experimental group without PDA, and there was no significant difference. The results showed that in the three-dimensional composite spheroid, the presence of PDA enhanced the cell activity to a certain extent. Based on the largely unchanged cell viability within the three-dimensional cell spheroid, the morphological changes in the cell spheroid incorporating different components of nanowires varied over time. This indicated that the PDA coating and the incorporation of strontium did not affect cell viability but instead influenced the interactions between the two kinds of cells within the three-dimensional composite spheroid, as well as the interactions between the cells and the material. The statistical results of the roundness and size of the three-dimensional cell spheroids serve as direct evidence to confirm these three kinds of interactions.

## 4. Conclusions

In this study, SrHAW with a high length–diameter ratio were prepared by hydrothermal synthesis, and their surfaces were coated with PDA to obtain rough surfaces for adsorbing macromolecules. The experimental results show that SrHAW can promote the formation of tubules, and the PDA coating on their surface has a positive effect on cell proliferation and differentiation in three-dimensional composite spheroids. SrHAW@PDA enhances cell–cell and cell–material interactions within composite spheroids, thereby regulating cellular activity and differentiation.

## Data Availability

Data will be made available on request.
